# Evaluation of Presepsin for Early Diagnosis of Sepsis in the Emergency Department

**DOI:** 10.3390/jcm14072480

**Published:** 2025-04-04

**Authors:** Andrea Piccioni, Silvia Baroni, Gloria Rozzi, Fabio Belvederi, Simone Leggeri, Fabio Spagnuolo, Michela Novelli, Giulia Pignataro, Marcello Candelli, Marcello Covino, Antonio Gasbarrini, Francesco Franceschi

**Affiliations:** 1 Department of Emergency Medicine, Fondazione Policlinico Universitario A. Gemelli-IRCCS, 00168 Rome, Italy; giulia.pignataro@policlinicogemelli.it (G.P.); marcello.candelli@policlinicogemelli.it (M.C.); marcello.covino@policlinicogemelli.it (M.C.); francesco.franceschi@unicatt.it (F.F.); 2 Università Cattolica del Sacro Cuore, 00168 Rome, Italy; silvia.baroni@unicatt.it (S.B.); gloria.rozzi01@icatt.it (G.R.); fabio.spagnuolo01@icatt.it (F.S.); michelanovelli5@gmail.com (M.N.); antonio.gasbarrini@unicatt.it (A.G.); 3 Unit of Chemistry, Biochemistry and Clinical Molecular Biology, Department of Laboratory and Hematological Sciences, Fondazione Policlinico Universitario Agostino Gemelli-IRCCS, Università Cattolica del Sacro Cuore, 00168 Rome, Italy; fabiobelvederi@gmail.com (F.B.); simone.leggeri1996@gmail.com (S.L.); 4 Medical and Surgical Science Department, Fondazione Policlinico Universitario Agostino Gemelli-IRCCS, Università Cattolica del Sacro Cuore, 00168 Rome, Italy

**Keywords:** sepsis, presepsin, molecular biomarkers, score and sepsis, ED

## Abstract

**Background**: to date, there are no specific markers available for diagnosing sepsis. Diagnosis is, indeed, mainly determined by clinical suspicion and the evaluation of the patient’s overall condition. This evaluation involves assessing various inflammatory markers, such as C-reactive protein (CRP) and procalcitonin (PCT), along with markers of tissue hypoxia, such as serum lactate. Additionally, it includes scores that account for complete blood count (CBC), organ function markers, and the patient’s vital parameters, including SOFA, qSOFA, SIRS, and NEWS. Over the years, various potential biomarkers have been studied; among these presepsin appears to offer some significant advantages. Objective: Presepsin, which is the N-terminal fragment of the soluble component of CD14, is primarily elevated in infectious conditions. Its levels rise much earlier in the context of infection compared to currently used biomarkers. As a result, Presepsin shows promise for the early identification of septic patients and could aid in prognostic assessment, allowing clinicians to prioritize care for critically ill individuals. **Methods**: this study aims to evaluate the role of serum presepsin in the early diagnosis of sepsis in patients who present to the emergency room with a clinical suspicion of sepsis. The secondary objectives include comparing the diagnostic performance of presepsin with traditional biomarkers currently used for sepsis diagnosis and assessing its utility as a prognostic biomarker for mortality risk stratification, in comparison with validated severity prediction scores. **Result**: Presepsin had valuable diagnostic utility for sepsis (AUC 0.946, *p* < 0.001) comparable to PCT (AUC 0.905, *p* < 0.001). **Conclusions**: the combination of Presepsin, PCT, and EWS yielded the highest diagnostic accuracy for sepsis.

## 1. Introduction

Sepsis is a widespread systemic disease and is a common reason for people to be admitted to the Emergency Department (ED). It is also one of the main causes of death among patients who access the ED [1]. 

According to the 2016 third international consensus on sepsis, sepsis is defined as a syndrome characterized by an uncontrolled response of the organism to an infectious stimulus, and it is considered a life-threatening condition [2,3]. The incidence of sepsis has been steadily increasing since the first consensus definition (Sepsis-1) in 1991, with approximately 49 million cases of sepsis and 11 million sepsis-related deaths all over the world in 2017 [4].

Based on existing literature, it is hypothesized that measuring presepsin levels in the ED could enhance the speed and specificity of diagnosis, compared to the current diagnostic approach, potentially improving outcomes for patients presenting with sepsis-like symptoms. The objective of our study is to evaluate the role of serum presepsin levels in the early diagnosis of sepsis in patients with suggestive clinical signs, comparing its performance with traditional biomarkers such as CRP, PCT, and blood cultures.

Septic shock is the most severe manifestation and should be defined as a subset of sepsis that presents particularly significant cellular, metabolic, and circulatory abnormalities. It is characterized by a drop in blood pressure, leading to reduced tissue perfusion pressure and causing characteristic shock-induced hypoxia. This condition results in an increase in mortality compared to sepsis alone [5].

Moreover, sepsis often presents a wide variety of symptoms that can resemble different medical conditions, highlighting the urgent need for more effective early diagnosis methods.

Over the years, scores (such as SOFA, qSOFA, SIRS, NEWS, and MEWS), have been developed by combining clinical and laboratory factors [6]: for instance, sepsis is clinically identified as a response to infection that causes organ damage such as to increase in the patient’s SOFA score by at least 2 points, compared to his baseline condition [7,8].

Septic shock is a subset of sepsis in which the serum lactate value is greater than 2 mmol/L despite the absence of hypovolemia, and in which the use of vasopressors is necessary to maintain the mean blood pressure at a level above 65 mmHg [9].

Biomarkers can offer a rapid and straightforward approach to aid in the early detection of sepsis in emergency departments [10,11]. Presepsin (sCD14-ST), in particular, is the 13 kDa N-terminal segment of CD14, a membrane glycoprotein, and co-receptor involved in the innate immune response constitutively expressed on the surface of monocytes and macrophages, even though it has been described in hepatocytes and Kupffer cells [12]. Presepsin (PSP) has been studied in several clinical settings as a biomarker of interest in infectious and renal failure conditions, although it is not integrated into international guidelines [13].

A significant increase in the release of Presepsin by peripheral monocytes is detectable at 2 h from the start of the infectious stimulus [14,15,16] and reaches its peak at 3 h [14,15]. It is also worth noting that presepsin levels vary depending on the type of infection, with a positive correlation with bacterial strains such as *Escherichia coli*, *Klebsiella*, *Pseudomonas*, *Streptococcus*, and *Acinetobacter* [17], as well as fungal infections [15,16]. For viral infections, the elevation of presepsin levels varies [17,18].

In peripheral mononuclear cells, exposure to lipopolysaccharide (LPS) resulted in an increase in median presepsin levels as early as 1 h after treatment. In a human monocytic cell line, presepsin was detected 1 h after LPS exposure, with levels peaking at 3 h [19]. The half-life of presepsin is 5 h [20]. The levels of presepsin show a gradual decrease in 7 days after admission [21].

Presepsin levels measured 24 and 72 h after hospital admission are more accurate for predicting the onset of septic shock than levels measured at admission [21]. Additionally, presepsin outperforms procalcitonin and C-reactive protein in predicting mortality among septic patients [22,23,24].

It has also been observed that presepsin may be an indicator of the development of renal failure and acute respiratory syndrome (ARDS) [22].

The significance of this biomarker is underscored by the diverse and complex clinical presentation of sepsis. In the context of sepsis, presepsin offers promise in the diagnosis, prognostic stratification of patients, and assessing the effectiveness of antibiotic therapy [23].

Presepsin is particularly effective in distinguishing sepsis from other non-infectious inflammatory conditions [19,25]. It exhibits similar sensitivity to procalcitonin (PCT) but boasts higher specificity, which leads to improved diagnostic accuracy. Research indicates that presepsin is not inferior to PCT in diagnosing sepsis [26,27,28,29] and, presepsin levels are found to be higher in patients with septic shock compared to those with sepsis. While presepsin is a less reliable predictor of bacteremia compared to procalcitonin (PCT), it performs better than the SOFA score, which is recommended by the Sepsis-3 consensus for identifying septic patients.

Presepsin has shown a strong predictive value for in-hospital mortality as well as for 28-day and 30-day mortality [30,31,32,33]. The most accurate predictions occur when measuring presepsin at baseline (time 0). While measurements taken at 1, 3, and 7 days are typically higher in non-surviving patients, they are not accurate in predicting mortality [34]. While research on the use of presepsin in the context of antibiotic stewardship is still in its early stages, it is showing promise [26,35].

## 2. Materials and Methods

We conducted a prospective observational cohort study in the adult Emergency Department of Policlinico Agostino Gemelli, University Hospital IRCCS in Rome. The study was carried out between May 2023 and October 2024. The study was performed in accordance with good clinical practice guidelines. A total of 216 patients were enrolled after providing written informed consent.

### 2.1. Inclusion Criteria

- Patients aged >18;
- Patients presenting to the emergency department with a clinical suspicion of sepsis;
- Signed written informed consent for participation in the study and for personal data processing.

### 2.2. Exclusion Criteria

- Age < 18 years;
- Pregnant women;
- Refusal to sign written informed consent for participation in the study and for personal data processing.

Patients were selected by the clinician during the initial assessment following their admission to the ED. During routine clinical practice blood tests, an additional venous blood sample was taken for the measurement of presepsin levels, beyond the standard diagnostic-therapeutic procedures for the patient.

Demographic and clinical data, results of blood/urine cultures, calculation of the score, serum presepsin levels, and other laboratory parameters (serum lactate, serum creatinine, CRP, PCT, complete blood count) were also collected. Presepsin levels were also evaluated through an additional k3-EDTA blood sample. These samples were immediately delivered to the Clinical Biochemistry laboratory, where after centrifugation, the obtained plasma was stored at −80 °C until measurement. Presepsin was measured by chemiluminescent immunoassay (CLEIA) using the TOSOH AIA 360 analyzer with the ST AIA-PACK Presepsin kit (Japan). The total precision of the method, including within-run, between-run, and within-days variations, was determined for three control levels, with an average CV of 4% for all analyzed levels.

We defined the following thresholds for positive and negative values:

○ For procalcitonin, a value below 0.5 ng/mL was regarded as negative. Patients with procalcitonin levels higher than 0.5 ng/mL were considered at risk of sepsis;
○ For presepsin, a negative value was defined as less than 165 pg/mL, and a positive value was greater than 600 pg/mL. Patients with presepsin levels higher than 165 pg/mL are patients with a high suspicion of sepsis.

The primary endpoint was the identification of sensitivity, specificity, and accuracy of the biomarker presepsin for the diagnosis of sepsis. The secondary endpoints were to compare the diagnostic performance of presepsin with that of procalcitonin and to evaluate the performance of the combination of these two variables in diagnosing sepsis.

The variables included in the study were summarized using descriptive statistical methods. Diagnostic accuracy was evaluated in terms of sensitivity, specificity, and positive and negative predictive values. Finally, ROC curves were constructed to assess the diagnostic accuracy of presepsin, and the Mann–Whitney test was performed.

The statistical analysis was performed using Medcalc. The present study has obtained the approval of the ethical board of our institution (number of approvals 0032518/23). The collected data were collected after having obtained written informed consent from the patient or a next of kin if the patient was incapacitated. Data were stored anonymously in a password-protected Excel file.

## 3. Results

**Figure 1:**
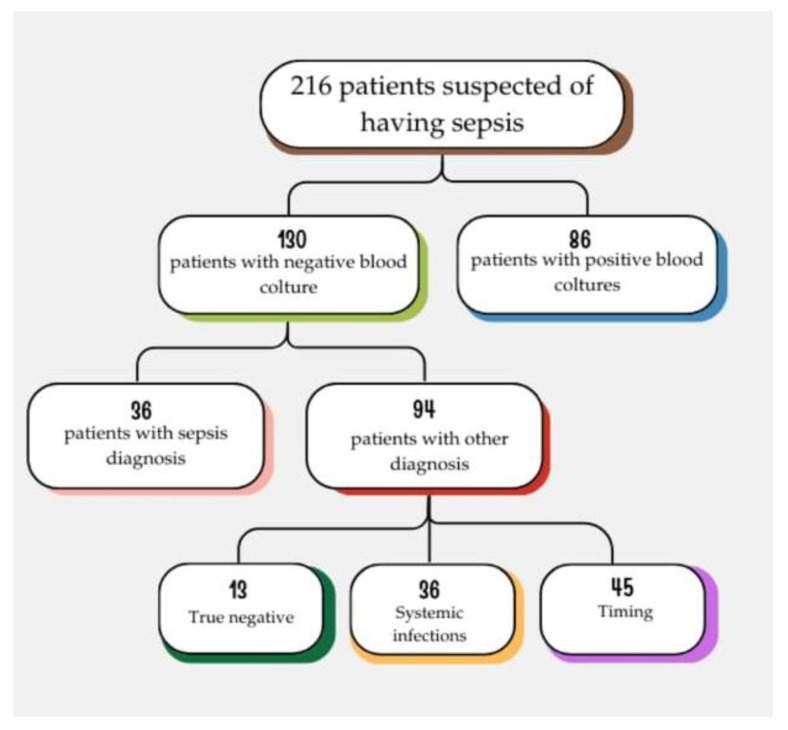
In these two groups of patients, we observed the behavior of the biomarkers we are studying to diagnose sepsis: procalcitonin and presepsin.

We enrolled 216 patients admitted to the ED, who were suspected of having sepsis. The patients enrolled in the following study were not undergoing treatment with either corticosteroids or immunosuppressive drugs. We divided the patients into two groups based on the results of the blood cultures: 86 patients had positive blood cultures (PBC) and were classified as true positives (TP), while 130 patients had negative blood cultures (NBC) [Figure 1].

Among the 86 patients with PBC, 89.5% had a presepsin level greater than 165 pg/mL, and 65.1% had a procalcitonin level exceeding 0.5 ng/mL. Notably, only 10.4% of these patients with PBC had a presepsin level at the infective threshold of 165 pg/mL, while 34.9% had a negative procalcitonin result.

**Figure 2:**
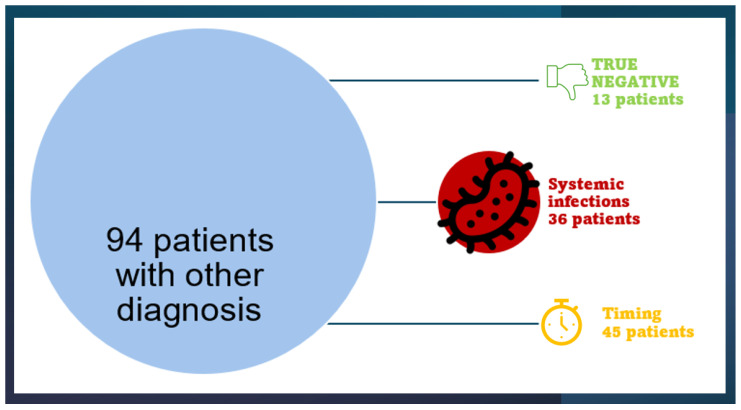
In this figure weshow that 94 patients were not diagnosed with sepsis at discharge from the emergency room, while 36 were diagnosed with sepsis.

In our assessment of patients with negative blood cultures, we examined the diagnoses made by physicians based on their clinical judgment, regarding the discharge diagnoses from the emergency department and subsequent admission to a ward. Out of 130 patients, 94 were not diagnosed with sepsis at discharge from the emergency room, while 36 were diagnosed with sepsis [Figure 2].

Of these 36 patients diagnosed with sepsis, 94.4% had a presepsin level exceeding 165 pg/mL, while 86.1% had a procalcitonin level above 0.5 ng/mL. Only 5.6% of this subgroup showed a negative result for presepsin, while 13.9% had a negative result for procalcitonin.

We conducted the Mann–Whitney test to compare patients with positive blood cultures to those with negative blood cultures who were diagnosed with sepsis. Our goal was to evaluate the values of presepsin and procalcitonin. The results indicated no significant differences between the two groups, with a *p*-value of 0.8383 for presepsin and a *p* value of 0.1688 for procalcitonin.

In the subgroup of patients with NBC, discharged from the emergency room with a diagnosis other than sepsis, 49 patients presented with both positive or negative presepin and procalcitonin. Among these, 13 patients had both presepsin and procalcitonin values negative, classifying them as true negatives (TN group). In contrast, 36 patients had procalcitonin levels exceeding 0.5 ng/mL and presepsin levels exceeding 165 pg/mL (systemic infection group). Additionally, there were 45 patients with discordant values for procalcitonin and presepsin (timing group). Of these, 6 patients had positive procalcitonin values and negative presepsin values, while 39 patients had positive presepsin values and negative procalcitonin values.

We performed the Mann–Whitney test to compare the group of patients with PBC and the group of patients classified as the “Timing group”. In this analysis, we found a statistically significant difference in the distributions of presepsin (*p* < 0.0001) and procalcitonin (*p* < 0.0001).

In this study, the Positive Predictive Value (PPV), Negative Predictive Value (NPV), sensitivity, and specificity were calculated as follows (Table 1):

- True Positives (TP): Patients with positive blood cultures and negative blood cultures but diagnosed with sepsis in the emergency department;
- False Positives (FP): Patients in whom the biomarker was elevated but who were not diagnosed with sepsis in the emergency department, along with patients in the timing group. It should be noted that in patients without a sepsis diagnosis in the emergency department, the diagnosis became positive for sepsis or systemic infection during hospitalization (thus, they are not strictly “false positives”);
- True Negatives (TN): Patients with both biomarkers negative and discharged without a diagnosis of sepsis or systemic infection, along with patients from the timing group where the biomarker was negative.

## 4. ROC Curves

*ROC Performance Curves of PSP and PCT in Blood Cultures*

**Figure 3:**
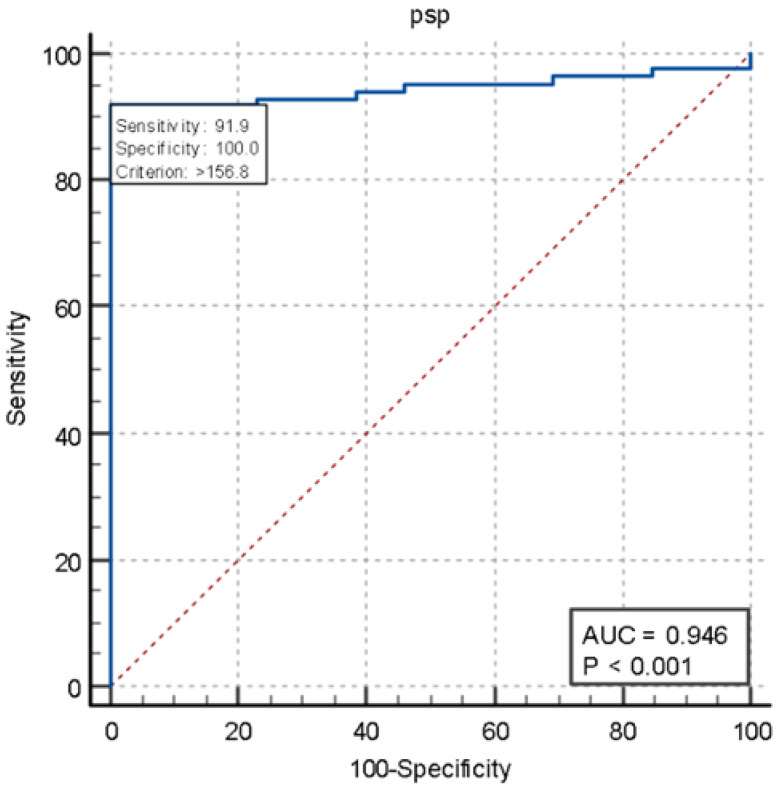
ROC of presepsin.

**Figure 4:**
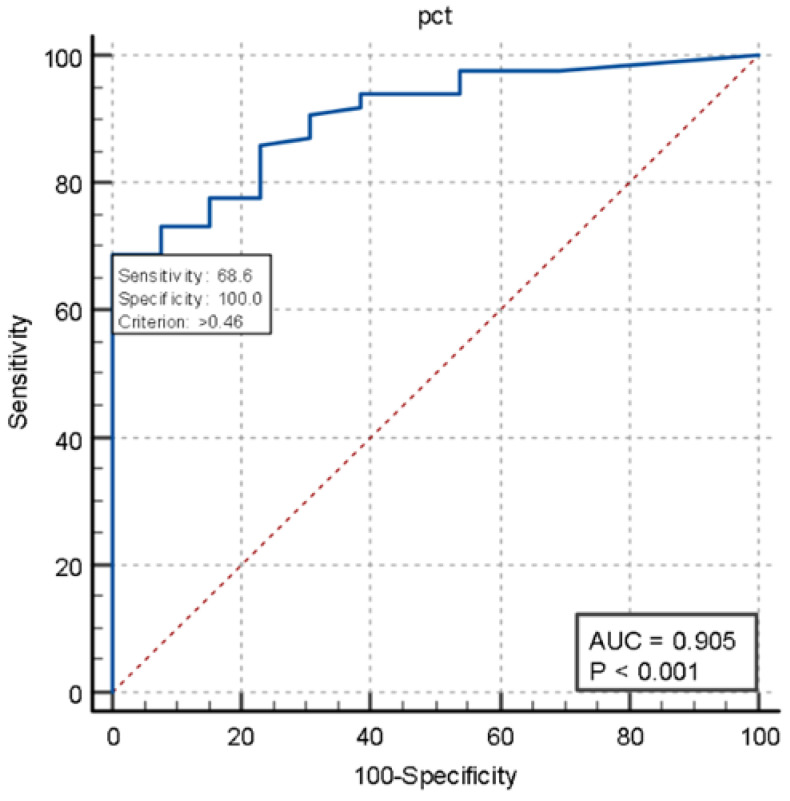
ROC curves of procalcitonin.

We also constructed ROC curves to assess the performance of presepsin (AUC 0.946, *p* < 0.001) and procalcitonin (AUC 0.905, *p* < 0.001) in two groups of patients: those with positive blood cultures (true-positive patients) and those with negative blood cultures who were not diagnosed with sepsis at discharge (true-negative patients). Presepsin showed 91.9% sensitivity while pct showed 68.6% sensitivity (Figure 3 and Figure 4). 

**Figure 5:**
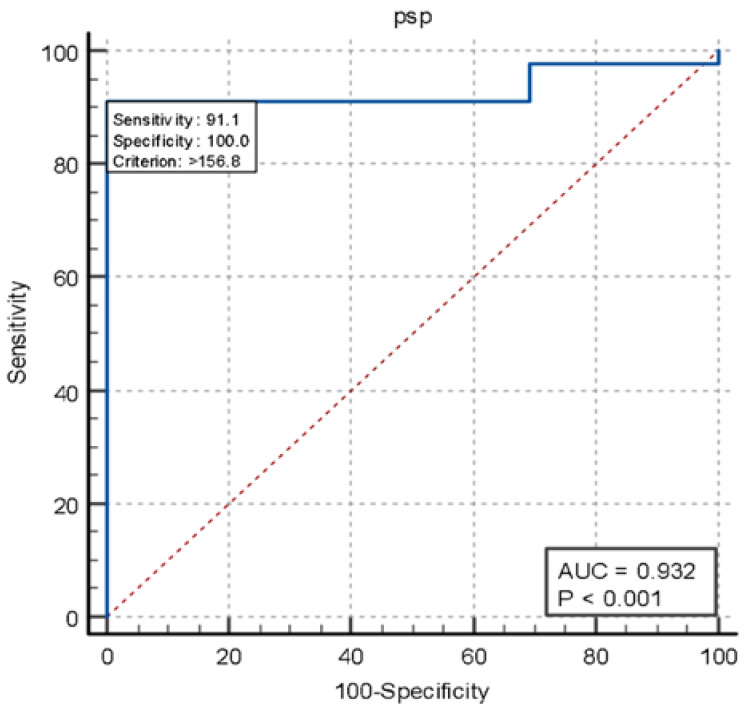
Curve ROC performance PSP in timing versus true negative.

**Figure 6:**
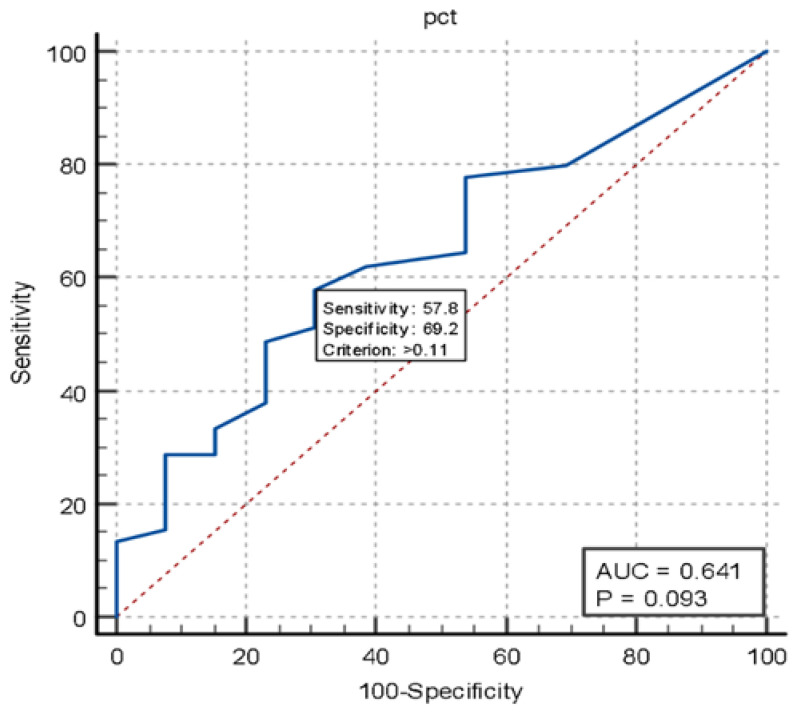
Curve ROC performance PCT in timing versus true negative.

In the timing and TN groups, presepsin demonstrated excellent sensitivity and specificity (91.1% sensitivity, AUC = 0.932, *p* < 0.001) compared to procalcitonin (57.8% sensitivity, AUC 0.641, *p* < 0.093) in the diagnosis of infected patients (Figure 5 and Figure 6).

## 5. Discussion

Sepsis is a heterogeneous disease that presents with highly variable clinical manifestations, especially in elderly patients and those with multiple comorbidities. The role of the emergency physician is crucial in the early recognition of sepsis, relying on vital parameters, clinical appearance, and scoring systems such as SOFA. Blood cultures are a crucial parameter in defining a septic patient, enabling us to identify the cause and administer appropriate antibiotic therapy. To confirm a sepsis diagnosis one of the key elements is performing a blood culture, ideally two sets, yet in almost 40% of all patients, they do not result positive, even though there is a clinical diagnosis of sepsis, supported by clinical presentation, scores and response to therapy [26].

Sepsis is a systemic condition that can present in a variety of ways and affect all organs and patient presentation can vary widely: for instance, while fever is the symptom most commonly associated with sepsis, it is also common to experience hypothermia or normothermia [28,29,36]. Arterial and venous vasodilation can occur, resulting in hypotension [37,38,39] and up to 60% of septic patients may experience depression of myocardial function, and a slight elevation in serum troponin levels is also common [38,39].

Patients with sepsis may experience rapid breathing (tachypnea) and shortness of breath (dyspnea) even when pneumonia is not present [37,40]. Signs of acute kidney injury, or oligo-anuria, can also occur irrespective of the primary infectious source, as a result of acute kidney injury induced by the septic process [40]. Septic patients may experience mental confusion, disorientation, hallucinations, changes in the sleep–wake cycle, and psychomotor agitation [41,42,43].

Overall, it is a complex condition to diagnose, and having some more tools in our bag to diagnose it, could help us and, ultimately, patients.

Based on our data analysis, a significant percentage of patients with positive blood cultures also exhibited elevated levels of procalcitonin and presepsin. Notably, presepsin demonstrated better accuracy than procalcitonin, as the number of false negatives was lower for presepsin. Specifically, there were only 9 patients with presepsin levels below 165 pg/mL, while 30 patients had procalcitonin levels below 0.5 ng/mL, even though they were then diagnosed with sepsis. It is also worth noting that, given its short half-life, it is likely that patients who had low levels of presepsin, even though septic, underwent the blood collection too late to capture its blood peak. Additionally, the combination of presepsin and procalcitonin was found to be effective in diagnosing sepsis, regardless of blood culture results. This is evidenced by the lack of significant statistical differences between the group with positive blood cultures and the group with negative blood cultures but a clinical diagnosis of sepsis.

According to the ROC curves in patients with positive blood cultures (septic), the sensitivity of presepsin is higher than that of procalcitonin, although both biomarkers are well correlated with the presence of sepsis. The identified cut-off values for these biomarkers indicate high negative predictive values, meaning that patients with positive blood cultures tend not to exhibit lower biomarker levels below the normal cut-off.

There is an ongoing debate around the right cut-off value for presepsin, with studies suggesting a range between 400 pg/mL and 864 pg/mL for identifying septic patients. A meta-analysis indicated that a range of 600 to 650 pg/mL maximizes Youden’s index for sepsis diagnosis [43].

Presepsin levels tend to be higher in elderly patients and increase with the severity of renal insufficiency. Proposed cut-offs for patients with renal impairment are set at 1000 pg/mL for creatinine levels above 1.5 mg/dL and 1300 pg/mL for levels exceeding 2 mg/dL. However, presepsin is not effective for diagnosing sepsis in patients with creatinine levels above 4 mg/dL [44]. Baseline presepsin values increase in patients with renal failure, proportionally to the decline in renal function. One study reported the following baseline presepsin ranges for patients, stratified by glomerular filtration rate (GFR): for patients with a GFR greater than or equal to 60 mL/min/1.73 m^2^, 60.8–85.9 pg/mL; for GFR 30–59 mL/min/1.73 m^2^, 68.7–150.0 pg/mL; for GFR 15–29 mL/min/1.73 m^2^, 117.0–200.0 pg/mL; and for GFR below 15 mL/min/1.73 m^2^, 1070.0–1400.0 pg/mL [45]. In patients with end-stage renal disease undergoing kidney transplantation, post-transplant presepsin levels are significantly lower than pre-transplant levels [45,46].

Presepsin concentrations increase proportionally to the degree of renal insufficiency, even in septic patients and elderly individuals.

Dialysis removes circulating presepsin, with hemofiltration being more effective than hemodialysis in clearing presepsin from circulation.

Specifically, it has been shown that dialysis achieving a β2-microglobulin clearance greater than 50 mL/min effectively removes presepsin, whereas dialysis with β2-microglobulin clearance below 30 mL/min does not; in such cases, pre-dialysis presepsin levels are predictive of post-dialysis levels [45]. It is also possible that presepsin is eliminated by renal replacement therapy techniques used in intensive care, like other sepsis markers. Therefore, in these patients, it is essential to consider the possibility that low presepsin levels may mask an underlying septic condition [46,47].

Plasma presepsin helps distinguish patients with acute pyelonephritis, using a cut-off of 340 pg/mL and in this context, urinary presepsin concentration has also proven useful, with a cut-off of 3650 pg/mL [48]. In septic patients, presepsin levels rise in cases of acute kidney injury due to sepsis and may predict its development; a cut-off of 572 pg/mL has been suggested for this purpose [49].

While presepsin is a less reliable predictor of bacteremia compared to procalcitonin (PCT), it performs better than the SOFA score, which is recommended by the Sepsis-3 consensus for identifying septic patients.

It is interesting to observe the behavior of these two biomarkers in the group of patients with negative blood cultures, without a clinical diagnosis of sepsis. We reassessed all these patients, including discharge diagnoses from the wards.

When both procalcitonin and presepsin levels exceed normal cutoffs simultaneously, this suggests a clinical picture of sepsis or an evolving systemic infection. This is supported by cases in which positive blood cultures indicate sepsis, as well as instances where blood cultures are negative, but sepsis is diagnosed in the emergency department or on the ward. If both biomarkers return negative results, sepsis can be ruled out, potentially indicating that the infection has been resolved or that therapy is effective.

Patients in the timing group provide further evidence of the complementary nature of these two biomarkers. First, we need to explain the meaning of the name “Timing”. We chose the term “Timing” for the group to highlight that the two biomarkers, presepsin and procalcitonin, exhibit different kinetics in the body, resulting in varying peak levels in the blood. This distinction allows us to gain valuable insights into the timing of a patient’s infectious status based on which biomarker is identified. Consequently, the presence or absence of each biomarker provides important information about the patient’s condition at the moment the measurement is taken.

When both markers move in the same direction, they enhance diagnostic value. On the other hand, when they are discordant they give us a lot of information. Patients with high levels of presepsin and low levels of procalcitonin likely indicate that the infectious process has just begun. This information allows for an early diagnosis of sepsis, enabling timely treatment to mitigate its consequences. In cases where patients have fluctuating presepsin levels along with low levels of procalcitonin, it is probable that the infection is localized. In contrast, patients with high procalcitonin levels and low presepsin levels have likely progressed beyond the early stage of infection, suggesting that they have already experienced a peak in their presepsin levels.

If presepsin has already peaked and declined, it may imply that the body’s immune system has already responded to the infection, but the persistence of high procalcitonin levels suggests that systemic inflammation is still ongoing. This could indicate the need for continued antimicrobial treatment, closer monitoring for signs of sepsis progression, and possibly adjunctive therapies such as anti-inflammatories. Furthermore, it highlights the importance of serial biomarker measurements to assess treatment efficacy and guide de-escalation strategies.

Based on the clinical presentation, additional diagnostic tests would be helpful to identify the source of the infection and initiate appropriate treatment.

Developing a standardized approach for this could be the focus of a future study.

## 6. Usefulness of Research

As previously explained, presepsin is a biomarker that can increase early in response to infections and sepsis, even before clinical signs become evident. Early detection allows clinicians to promptly initiate antibiotic therapy, thereby reducing the risk of disease progression and improving survival rates.

Moreover, presepsin can assist in differentiating sepsis from other inflammatory conditions that may not require immediate antibiotic intervention. According to our results in cases where presepsin and pct levels are normal or low, the diagnosis of sepsis can be excluded with a high degree of certainty, thus reducing the need for unnecessary treatments and minimizing the risk of side effects or antibiotic resistance.

Presepsin levels are correlated with the severity of sepsis and the appropriateness of antibiotic therapy. By monitoring presepsin, clinicians can assess the intensity of the inflammatory response and determine the risk of severe outcomes, such as severe sepsis or septic shock, facilitating the personalization of treatment. It is evident that its measurement, combined with clinical symptoms and other diagnostic tests, supports more informed decisions regarding treatment, monitoring, and management of sepsis.

Currently, presepsin can be measured using the PATHFAST Presepsin point-of-care system directly in the emergency department [50], with results available within 15 min. Alternatively, traditional laboratory assays can be used, providing results in 1–2 h.

All of this results in the optimization of healthcare resources and a reduction in the number of hospital admissions or length of hospital stays.

## 7. Limitations

Our study is a pilot survey investigating the role of presepsin as a biomarker for the early diagnosis of sepsis in patients who present to the emergency room with a clinical suspicion of the condition. Although our sample size is limited, the results provide interesting insights for further research. However, previous studies with larger sample sizes [46,47,51] have demonstrated that presepsin can be a valid biomarker for the early diagnosis of sepsis, reinforcing the interest in its clinical application.

Our study examined cases received at the DEA of a second-level university hospital in a metropolitan city. To confirm our findings, a multicenter study involving Level I Emergency Departments and Emergency-Urgency Departments in both urban and rural settings, as well as university and non-university hospitals, would be necessary. Additionally, we conducted a single-point assessment of presepsin values. Given its rapid kinetics, serial measurements every 4–6 h within the first 24 h would allow for an evaluation of the biomarker’s dynamic trend, positioning the patient in different phases of the septic process.

In this pilot study, we have not yet considered the cut-off of presepsin in patients with renal insufficiency. However, with a larger sample size, we will address this important parameter in our ongoing research.

Finally, we did not assess the impact of presepsin on the prognostic stratification of septic patients or its role in guiding the discontinuation of antibiotic therapy. Masson et al., in a multicenter randomized study involving 997 patients, demonstrated a decrease in presepsin levels seven days after the initiation of effective antibiotic therapy [52]. This is certainly an interesting point that we could investigate in future studies, but now, as this study focuses on the early diagnosis of sepsis, we have concentrated on emphasizing the important role of presepsin as a biomarker for early diagnosis in the emergency department.

## 8. Conclusions

The early and accurate diagnosis of sepsis is crucial for ensuring that patients receive timely and effective treatment. Currently, research is focused on identifying new biomarkers that can facilitate rapid and precise diagnosis, ultimately improving prognosis and clinical outcomes.

Our study has shown that the combined use of two biomarkers, presepsin, and procalcitonin, is highly effective in diagnosing sepsis. Both biomarkers also demonstrate a high negative predictive value in this context. Integrating these biomarkers can enhance diagnostic precision, particularly in cases where clinical presentations are ambiguous or when early diagnosis is essential. This dual approach may provide emergency physicians with a more reliable toolkit for the rapid identification of sepsis, potentially improving patient outcomes through earlier and more targeted interventions.

Furthermore, presepsin has been shown to have greater sensitivity and specificity than procalcitonin for the early diagnosis of sepsis or localized infections, making it an excellent tool for use in emergency situations.

## Figures and Tables

**Table 1:** VPP, VPN, SENSITIVITY AND SPECIFICITY.

TP psp: 111	FP psp: 36 + 39	PPV psp: 111/(111 + 36 + 39) = 59.6% NPV psp: 13 + 6/(13 + 6 + 11) = 63.3%
TN psp: 13 + 6	FN psp: 11	Sens psp: 111/(111 + 11) = 90.98% Spec psp: 13 + 6/(13 + 6 + 36 + 39) = 20.2%
TP pct: 87	FP pct: 36 + 6	PPV pct: 87/(87 + 36 + 6) = 67.4% NPV pct: 13 + 39/(13 + 39 + 35) = 59.7%
TN pct: 13 + 39	FN pct: 35	Sens pct: 87/(87 + 35) = 71.31% Spec pct: 13 + 39/(13 + 39 + 36 + 6) = 55.3%

## Data Availability

The articles cited in this paper are available on PubMed^®^, UptoDate^®^, and Cochrane^®^.
